# Trueness of full-arch IO scans estimated based on 3D translational and rotational deviations of single teeth—an in vitro study

**DOI:** 10.1007/s00784-021-04309-5

**Published:** 2021-11-27

**Authors:** Johanna Radeke, Annike B. Vogel, Falko Schmidt, Fatih Kilic, Stefan Repky, Jan Beyersmann, Bernd G. Lapatki

**Affiliations:** 1 Department of Orthodontics, Ulm University, Albert-Einstein-Allee 11, 89081 Ulm, Germany; 2 Institute of Statistics, Ulm University, Helmholtzstr. 20, 89081 Ulm, Germany

**Keywords:** Intraoral scanning, Digital impression, Full arch scan, Trueness

## Abstract

**Objectives:**

To three-dimensionally evaluate deviations of full-arch intraoral (IO) scans from reference desktop scans in terms of translations and rotations of individual teeth and different types of (mal)occlusion.

**Materials and methods:**

Three resin model pairs reflecting different tooth (mal)positions were mounted in the phantom head of a dental simulation unit and scanned by three dentists and three non-graduate investigators using a confocal laser IO scanner (Trios 3®). The tooth-crown surfaces of the IO scans and reference scans were superimposed by means of best-fit alignment. A novel method comprising the measurement of individual tooth positions was used to determine the deviations of each tooth in the six degrees of freedom, i.e., in terms of 3D translation and rotation. Deviations between IO and reference scans, among tooth-(mal)position models, and between dentists and non-graduate investigators were analyzed using linear mixed-effects models.

**Results:**

The overall translational deviations of individual teeth on the IO scans were 76, 32, and 58 µm in the lingual, mesial, and intrusive directions, respectively, resulting in a total displacement of 114 µm. Corresponding rotational deviations were 0.58° buccal tipping, 0.04° mesial tipping, and 0.14° distorotation leading to a combined rotation of 0.78°. These deviations were the smallest for the dental arches with anterior crowding, followed by those with spacing and those with good alignment (*p* < 0.05). Results were independent of the operator’s level of education.

**Conclusions:**

Compared to reference desktop scans, individual teeth on full-arch IO scans showed high trueness with total translational and rotational deviations < 115 µm and < 0.80°, respectively.

**Clinical relevance:**

Available confocal laser IO scanners appear sufficiently accurate for diagnostic and therapeutic orthodontic applications. Results indicate that full-arch IO scanning can be delegated to non-graduate dental staff members.

## Introduction

The development of chairside intraoral (IO) scanners started with techniques for scanning small arch segments for single-tooth restorations [1]. In recent years, the technology used in IO scanners has developed rapidly, and full-arch scans—as usually required in orthodontics—are a common feature of new-generation systems. Direct generation of virtual, three-dimensional (3D) models of the dental arches has several benefits, including the immediate availability of the model; this enables the chairside visualization and evaluation of the patient’s intraoral situation, and the laboratory process for stone-model fabrication can be omitted. In addition, virtual models can be shared easily among professionals at no additional cost or risk of damage. Although using direct digital models avoids several sources of inaccuracy of the indirect approach—such as impression-taking and fabrication of stone casts—it can involve other, new sources of error. More specifically, full-arch IO scans are based on consecutively recorded images of small dental-arch segments, which requires accurate superimposition of overlapping surfaces. It remains unclear whether, and to what extent, the accuracy of this procedure is affected by individual morphological factors such as dental anatomy and tooth malpositions.

ISO 5725–1 defines the accuracy of measurement methods and results in terms of trueness and precision. For IO scanning, trueness describes how closely the virtual dental arch model resembles the physical model—i.e., the intraoral situation—whereas precision measures consistency among virtual models obtained by repeated IO scans [2]. Most previous studies describe deviations between digital full-arch models created by different scanning methods as linear distances between surface meshes after the closest point superimposition, whereas the directions of the deviations are evaluated visually based on color-coded images [3–10]. Alternatively, deviations from defined landmarks on crowns have been used to compare IO scans with desktop scans. These studies also address clinical questions concerning the effects of the following: operator’s experience [7, 11], scanning strategy [4, 12, 13], tooth malpositions [4], and arch width [5]. It must be noted that the abovementioned approaches are unable to specify the deviation of single teeth in all six degrees of freedom, i.e., in terms of 3D translation and rotation [14]. This is important, because deviations of individual teeth and tooth movements are usually described in such terms—in the field of orthodontics, for example. Hence, these terms of description could also be useful for evaluating virtual dental arch models.

The aim of this in vitro study was to assess the trueness of IO scans of three pairs of resin models reflecting different types of tooth (mal)positions. Desktop scans of these resin models were used to obtain a virtual reference model with high trueness for comparison. The deviations between IO scans and desktop scans were evaluated with respect to the 3D translational and rotational deviations of individual teeth. Also examined was the effect of the operator’s level of education on the trueness of IO scans.

## Materials and methods

### Dental-arch models investigated

**Fig. 1:**
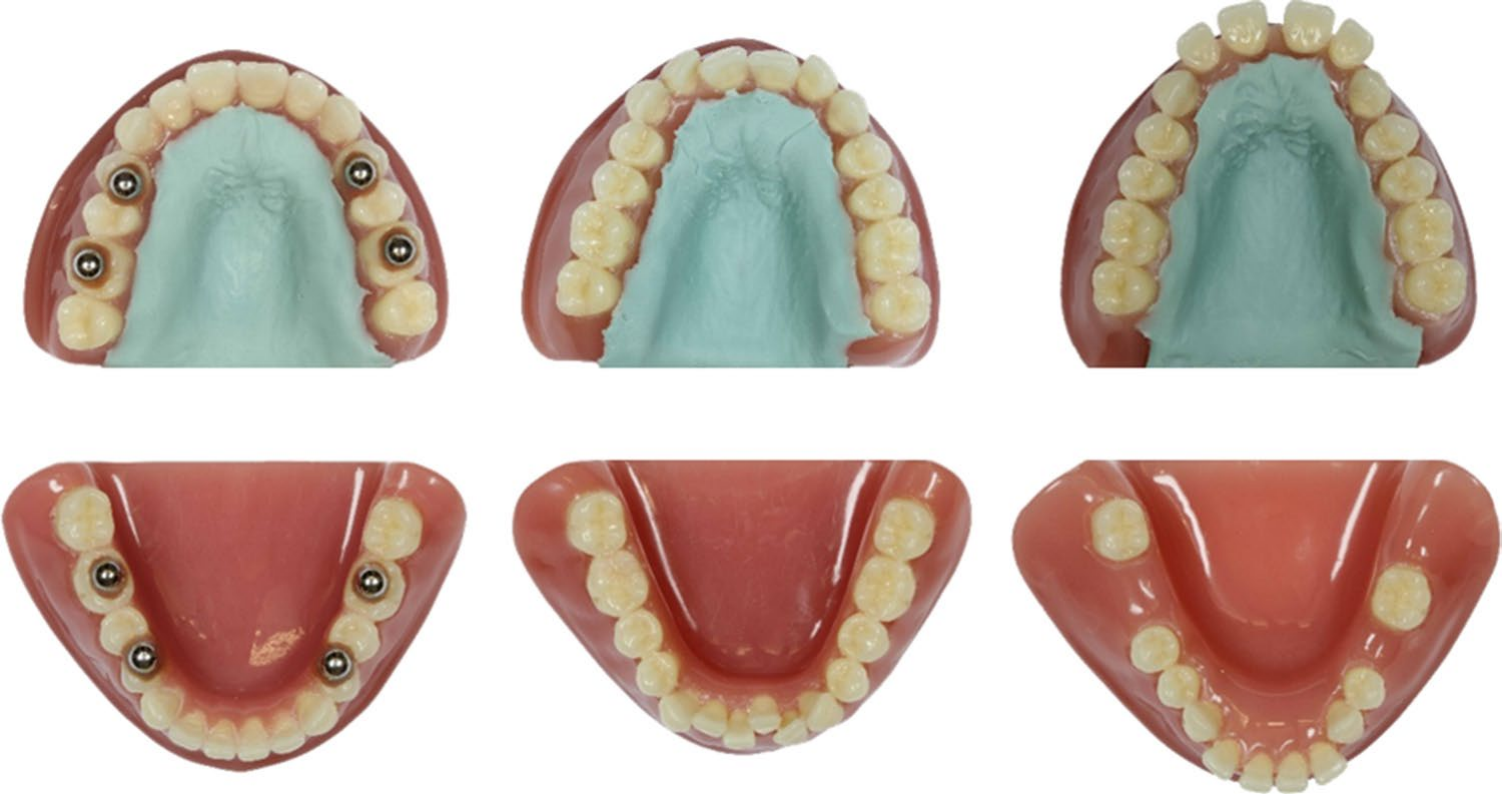
Investigated model pairs. **A** Ideal dental arches without tooth malpositions. **B** Models showing moderate to severe anterior crowding with deviations of anatomical contact points between 0.8 and 3 mm leading to a Little’s irregularity index of 10.5 and 9.8 for the upper and lower arch, respectively. **C** Models with flared incisors and interdental spaces ranging from 0.2 to 2.5 mm; three lateral teeth are missing to simulate edentulous regions

Three sets of resin models of the upper and lower dental arches were used as physical models. Each model represented one of three oral situations (Fig. 1): (A) an ideal dental arch without tooth malpositions, (B) anterior crowding, and (C) flared incisors combined with interdental spaces and three missing lateral teeth [15]. The models were fabricated using denture teeth (Bioplus® for upper and lower anterior teeth and Genios® for upper and lower posterior teeth, both DeguDent GmbH; Hanau-Wolfgang, Germany) and denture resin (Palapress, Heraeus Kulzer; Hanau, Germany). Basically, the resin models were those used in two previous studies [15, 16]. For the present study, however, the maxillary models were equipped with a palatal surface made of silicone including palatal rugae to obtain a more realistic morphology (Fig. 1).

### Digital reference models

To obtain accurate digital reference models, the physical models were optically scanned using a desktop scanner (d-Station3D, Breuckmann GmbH; Meersburg, Germany) specifically designed for dental casts or impressions. This structured light scanner uses active triangulation with a predefined triangulation angle of 20°. It features a camera and a rotary-swivel unit moving to different positions for single scans. Each of the scans covers the entire object from a single sensor position. As recommended by the manufacturer, each scanning procedure included 15 single scans which were automatically combined by the scanning software. In accordance with the definitions in standard 2634/2 of the Association of German Engineers (VDI), the scanner has a maximum deviation of ± 20 µm as specified by the manufacturer. Antiglare spray (Zir24, FZ3D; Hamburg, Germany) was used to create an anti-reflective coating on the acrylic model surfaces, resulting in a surface-layer thickness of 15,5 ± 2,6 µm as determined in a previous study [15]. Reference scans from each model were performed only once by one person with great experience with this procedure (FK).

### Intraoral scans

IO scans of the three different models were generated by six investigators (three dentists and three non-graduate investigators) using a Trios 3 Color IO scanning system (3Shape; Copenhagen, Denmark). This IO scanner is specified by the manufacturer by a trueness of 6.9 ± 0.9 µm and a precision of 4.5 ± 0.9 µm. All investigators were trained in the handling of the Trios 3 scanner prior to the study, had a similar level of intraoral scanning experience, and received standard instructions for performing the scans in the dental simulation units. Each resin model was mounted in a phantom head with torso (Adam™ dental patient simulator with G40 jaw simulator and rubber facemask, KaVo; Biberach an der Riss, Germany) that was positioned on a dentist’s chair. The ideal arch model (A) was scanned three times by each investigator to enable evaluation of intra- and inter-examiner reproducibility between repeated IO scans, whereas the two malocclusion models (B, C) were only scanned once. As recommended by the manufacturer, each scan followed a prescribed path that started on the occlusal surface of the right second molar [4, 12].

### Data processing and analysis

**Fig. 2:**
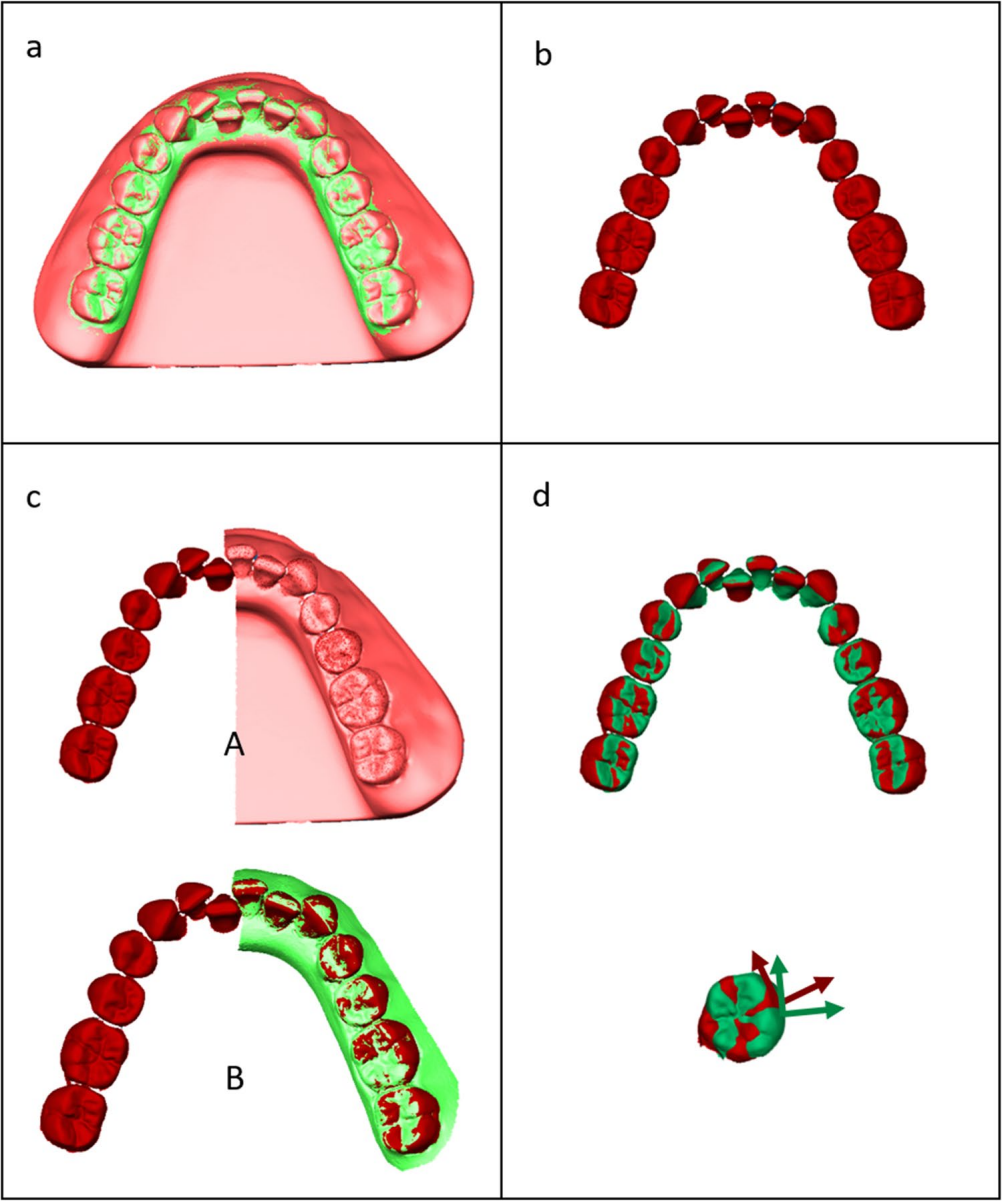
Superimposition technique used to determine three-dimensional deviations of intraoral (IO) scans from reference scans in terms of individual tooth positions. Preparation of necessary components. **a** Step 1: superimposition of tooth-crown surfaces of intraoral scan (green) and reference desktop scan (pink) by means of best-fit alignment, selecting all teeth for alignment. **b** Step 2: separation of individual tooth crowns from IO scan and determination of facial axis (FA) points. **c** Step 3: positioning of separated tooth crowns on the complete reference model (A) and IO-scanned model (B) by means of best fit. **d** Step 4: spatial evaluation of differences between corresponding coordinate systems. Calculation of single-tooth crown deviations at FA points expressed in translations along and rotations around the three spatial axes. Tooth-related coordinate systems originating at the FA points are clockwise in the first and third quadrants and counterclockwise in the second and fourth quadrants

We did not apply any post-processing to the IO scans and reference scans. The tooth-crown surfaces of each digital model derived from the IO scans were superimposed onto the tooth-crown surfaces of the corresponding reference model using a best-fit algorithm implemented in the OptoCat® software (version 11.01.06, AICON 3D Systems; Braunschweig, Germany) by selecting all teeth for alignment and applying a search range of 0.1 mm (Fig. 2a). The maximum number of iterations was 30. We then separated the crown surfaces of each tooth from one IO scan of each model type (Fig. 2b). On each separated crown, we then automatically determined the facial axis (FA) point using the OnyxCeph software (OnyxCeph^3^™; Chemnitz, Germany). The procedure of dental arch model segmentation was performed only once per tooth. It included automatic detection of the corresponding tooth crown margin, which allowed manual correction in cases where the software obviously identified the gingival margin incorrectly (such problem mainly occurred at interproximal regions). At the origin of the FA point, we implemented a coordinate system pointing in the mesial (x), buccal (y), and extrusive (z) directions [17].

The separated individual crown surfaces with fixed FA points and linked tooth coordinate systems were superimposed onto both the reference models (desktop scans) and IO scans by means of best-fit alignment (Fig. 2c). The following step was the evaluation of the spatial differences between the positions and orientations of corresponding coordinate systems in the superimposed reference and IO scans. This enabled the determination of differences in individual tooth positions expressed as (a) translations along and (b) rotations around the three spatial axes (Fig. 2d). All data processing and analysis were performed by one single investigator (FK).

#### Statistical analyses

We used linear mixed-effects modelling to statistically evaluate the deviations of the IO scans from the desktop scans. To account for the effects of different raters and repetitions, those were included in the model as random effects. The other variables—namely tooth, tooth type, and type of tooth (mal)position—were included in the underlying model according to the information of interest. This resulted in a separate model for each subject of analysis:

1. To give an overall impression of how the IO scan deviated from the reference, we also included all variables of interest as random effects in our statistical model.
2. To take account of the single teeth, we calculated one model for each single tooth and classified the setting—i.e., the three models of different tooth (mal)positions—as well as raters and repetitions as random effects.
3. The effect of different tooth (mal)positions was estimated by considering the teeth as a random effect and estimating the fixed effect of the underlying setting.
4. Using the repeated IO scans from the ideal dental-arch model, we estimated the differences in the position of the individual teeth among the three scans performed by one investigator (i.e., intrarater variability) by calculating standard deviations (*SD*) for the results of the three repeats separately for each investigator and tooth. These *SD* were then combined using a simple linear model to determine overall variation and to calculate 95% confidence intervals.
5. Accordingly, differences in the position of the individual teeth among scans performed by different investigators (i.e., interrater variability) were estimated by calculating *SD* for the results of all six investigators separately for each repeat and tooth. Also, these *SD* were then combined using a simple linear model to characterize overall variation.
6. The effect of education—i.e., the difference between dentists and non-graduate investigators—was estimated by integrating the type of rater as a fixed effect in a mixed-effect model.

## Results

### Estimated 3D deviations of IO scans from reference desktop scans—overall effect

**Fig. 3:**
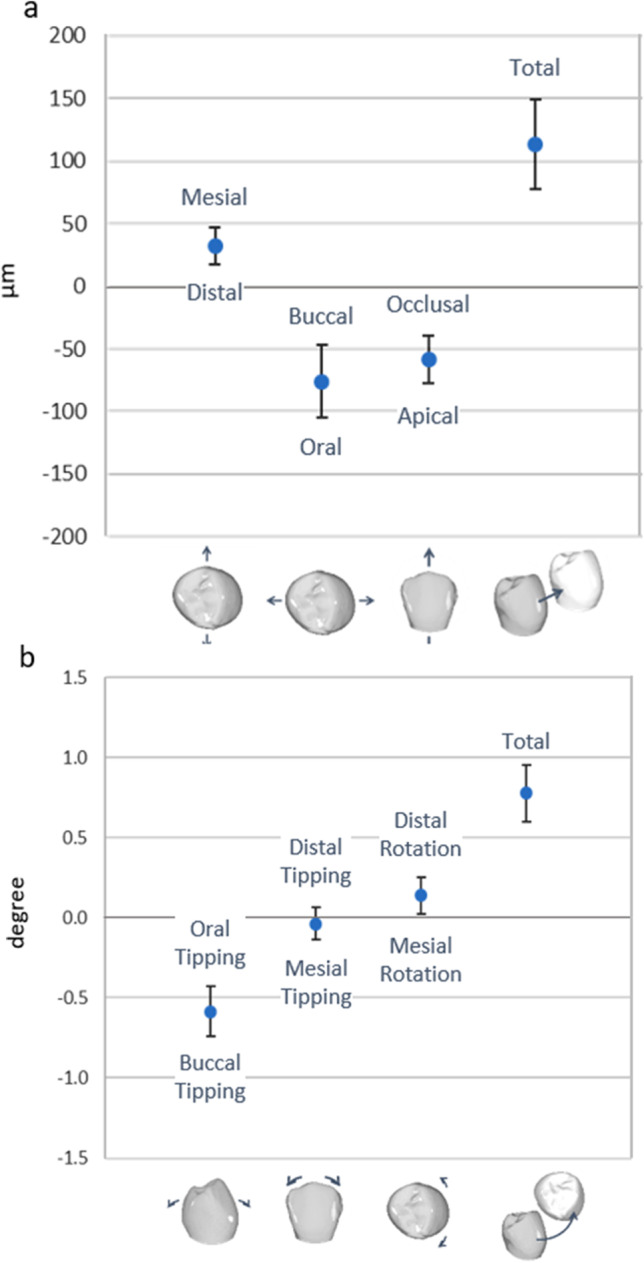
Translational (**a**) and rotational (**b**) deviations pooled for all teeth, models, raters, and repetitions, which were integrated in the statistical model as random effects. Values are plotted with 95% confidence interval to estimate clinical relevance of the calculated deviations

Figures 3a and 3b show the 3D components of the translational and rotational deviations of the IO scans and the estimated overall effect, i.e., pooled over all teeth, models, raters, and repetitions. Most deviations were quite small, with translations of 32, 76, and 58 µm in the mesial, oral, and apical directions, respectively, resulting in a total displacement of 114 µm (Fig. 3a). Ninety-five percent confidence intervals ranged from 29 µm (mesio-distal) to 59 µm (bucco-oral). Rotational deviations of 0.58° buccal tipping, 0.04° mesial tipping, and 0.14° distorotation of the tooth crowns were observed, resulting in a combined rotation of 0.78° (Fig. 3b). Here, 95% confidence intervals ranged from 0.20° (mesio-buccal tipping) to 0.31° (bucco-oral tipping).

### 3D translational deviations of individual teeth on IO scans

**Fig. 4:**
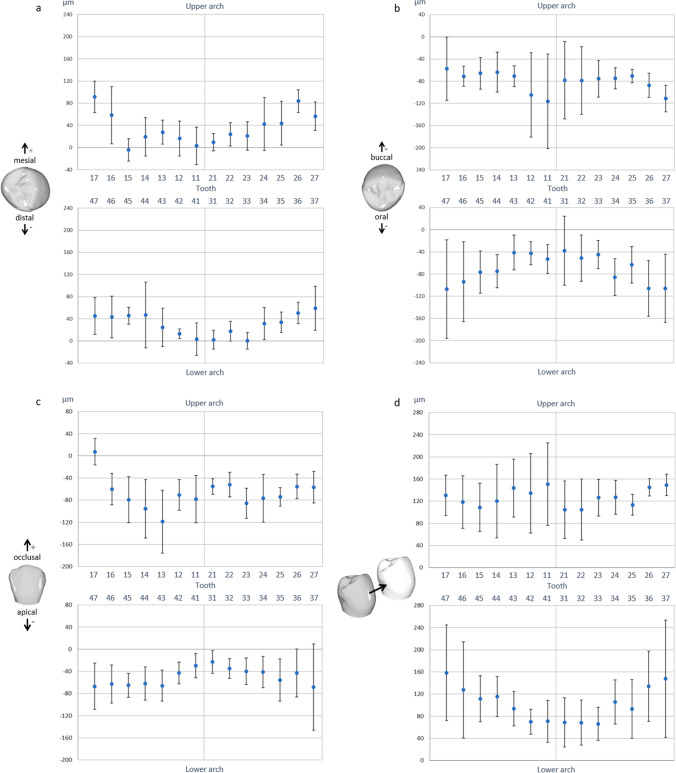
Translational deviations for individual teeth in the upper and lower arches, differentiated according to the three spatial axes (**a**–**c**) and total displacement (combined three-dimensional displacement; **d**). Models, raters, and repetitions were considered random effects. Values are plotted with 95% confidence interval to estimate clinical relevance of the calculated deviations. Statistically significant differences between deviations for different teeth are indicated by non-overlapping confidence intervals

**Fig. 5:**
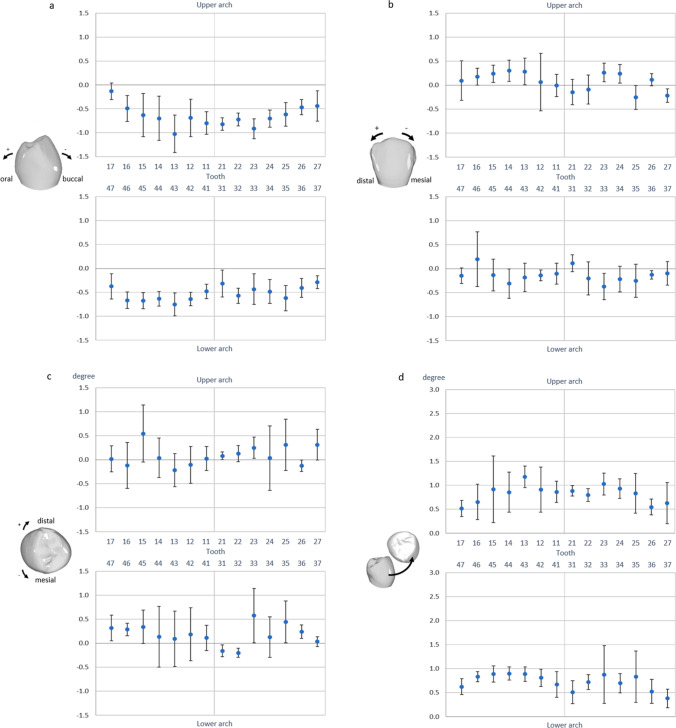
Rotational deviations for single teeth in the upper and lower arches, differentiated according to the three spatial axes (**a**–**c**) and total displacement (combined three-dimensional deviation; **d**). Models, raters, and repetitions were considered random effects. Values are plotted with 95% confidence interval to estimate clinical relevance of the revealed deviations. Statistically significant differences between deviations for different teeth are indicated by non-overlapping confidence intervals

Figures 4 and 5 show the 3D components of translational and rotational deviations, respectively, of IO scans from reference desktop scans for different tooth types. The data for the six investigators and three (mal)position models were pooled.

Analysis of the mesio-distal translational displacements (Fig. 4a) revealed a trend toward an increasingly mesial displacement from the anterior to the posterior teeth of both jaws. Accordingly, displacements of up to 91 µm from the reference scans were observed for molars and premolars, whereas deviations for incisors and canines were close to zero (max. 24 µm).

In the bucco-oral direction (Fig. 4b), IO scans showed individual teeth to be orally displaced, as indicated by the negative values of these deviations. The individual teeth in the upper arch did not exhibit a systematic pattern with regard to the extent of deviation, with estimated values ranging between -57 and -116 µm. In the lower arch, however, deviations were found to increase from the central incisors (-38 µm) to the second molars (-107 µm).

In the vertical dimension (Fig. 4c), individual teeth on IO scans showed a clear tendency toward intrusive displacement. Corresponding deviations were smaller in the lower arch (-23 to -68 µm) than in the upper arch (8 to -119 µm) and tended to be smaller for anterior teeth than for posterior teeth, except for tooth 17.

The resulting translational deviation (Fig. 4d) was the largest for the lower right molars (158 µm) and the smallest for the lower front teeth (66 µm). Ninety-five percent confidence intervals for the total translations were the largest for the lower second molars (212 µm, tooth 37) and the smallest for tooth 26 (31 µm).

### 3D rotational deviations of individual teeth on IO scans

Generally, rotational deviations of individual tooth positions on the IO scans were quite small (Fig. 5). In addition, we could not find any systematic patterns for the upper or lower dental arch for any rotational direction. The largest deviations were observed for the rotational component around the mesio-distal axis (Fig. 5a), with tooth crowns on IO scans generally tipped slightly buccally (range: -0.13 to -1.02°). Such buccal tipping was most pronounced in the upper canines and the lower right canine and premolars. Rotational deviations around the bucco-oral axis (Fig. 5b), signifying mesio-distal tipping of the crown, ranged from -0.37 to 0.30°. Rotational deviations around the vertical axis (Fig. 5c), indicating mesio-distal rotation of the crown, were somewhat larger (range: -0.22 to 0.58) and had greater variability. The 95% confidence interval was the smallest for tooth 36 (0.17° mesio-distal tipping; Fig. 5b) and the largest for tooth 24 (1.34° mesiorotation; Fig. 5c).

### 3D deviations of individual teeth in tooth (mal)position models, with pooled data for individual teeth

**Fig. 6:**
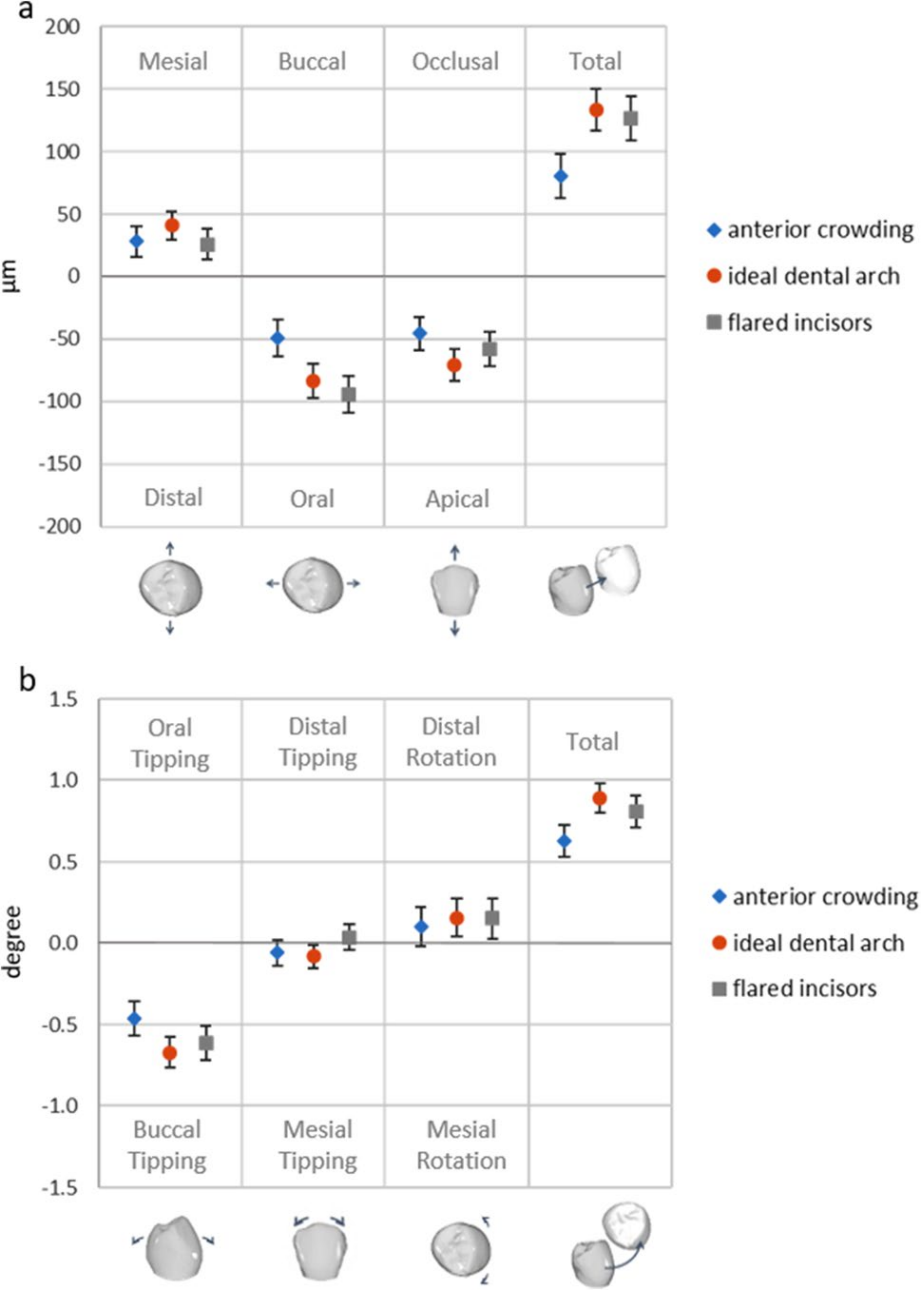
Translational (a) and rotational (b) deviations in models with different malpositions. Single teeth, raters, and repetitions were considered random effects. Values are plotted with 95% confidence interval to estimate clinical relevance and significance of differences. Statistically significant differences between models are indicated by non-overlapping confidence intervals

With respect to differences among the three models regarding individual tooth deviations, the largest translational deviations were recorded for the ideal dental-arch model (model A) except for bucco-oral displacements (*p* < 0.001), which were the largest in the model with flared incisors (model C, Fig. 6a). Translational deviations in the anterior crowding model (B) were significantly smaller than in the other two models (*p* < 0.001), mainly because the deviation in the bucco-oral direction was significantly smaller (*p* < 0.001). Regarding rotational components, the largest deviations were observed for the ideal dental-arch model, around all three spatial axes (Fig. 6b). Deviations differed significantly (*p* < 0.001) between the anterior crowding model and ideal dental-arch model; this was due to a significantly smaller amount of oral tipping in the anterior crowding model. Patterns were similar in the upper and lower arches.

### Intra- and interrater reliability

Regarding translational deviations within the three scans of the ideal dental-arch model performed by one rater, *SD* and *CI* were 20 ± 2 µm in the mesio-distal direction, 33 ± 5 µm in the bucco-oral direction, 28 ± 3 µm in the apico-occlusal direction, and 37 ± 5 µm for the resulting total translation. With respect to rotational deviations, *SD* and *CI* were 0.19 ± 0.02° for bucco-oral tipping, 0.13 ± 0.01° for mesio-distal tipping, 0.17 ± 0.02° for mesio-distal rotation, and 0.17 ± 0.02° for the resulting total rotation (Table 1).

**Table 1:** Intra- and interrater variations as estimated by standard deviations (*SD*) and 95% confidence intervals (CI) for the corresponding translational and rotational deviations of individual tooth positions

Translational deviations (*n* = 504)	Intrarater variations (*SD*) value ± *CI* [µm]	Interrater variations (*SD*) value ± *CI* [µm]
Mesio-distal	20 ± 2	28 ± 2
Bucco-oral	33 ± 5	48 ± 7
Apico-occlusal	28 ± 3	40 ± 4
Total	37 ± 5	53 ± 7
Rotational deviations (*n* = 504)	Intrarater variations *SD* value ± *CI* [°]	Interrater variations (*SD*) value ± *CI* [°]
Bucco-oral tipping	0.19 ± 0.02	0.25 ± 0.03
Mesio-distal tipping	0.13 ± 0.01	0.18 ± 0.01
Mesio-distal rotation	0.17 ± 0.02	0.24 ± 0.02
Total	0.17 ± 0.02	0.25 ± 0.03

The *SD* and *CI* among single scans performed by the six raters for translational displacements were 28 ± 2 µm in the mesio-distal direction, 48 ± 7 µm in the bucco-oral direction, 40 ± 4 µm in the apico-occlusal direction, and 53 ± 7 µm for the resulting total translation. For rotational displacements, corresponding *SD* and *CI* were 0.25 ± 0.03° for bucco-oral tipping, 0.18 ± 0.01° for mesio-distal tipping, 0.24 ± 0.02° for mesio-distal rotation, and 0.25 ± 0.03° for the resulting total rotation.

### Evaluation of differences between dentists and non-graduate investigators

Comparison of the IO scans performed by dentists and non-graduate investigators revealed smaller deviations from the reference desktop scans for the IO scans performed by non-graduate investigators. This difference was statistically significant for both, the translational and rotational vectors (*p* < 0.05). The corresponding quantitative estimates for this difference ranged between 6 and 15 µm for the three translational components, and between 0.03 and 0.11° for the three rotational components (Table 2).

**Table 2:** Differences between intraoral scans of graduated and non-graduated dental staff as estimated by linear mixed-effects model. Negative signs indicate smaller deviations in the scans of non-graduated staff. CI: 95% confidence intervals

Translational deviations (*n* = 822)	Difference between graduated and non-graduated dental staff value ± *CI* [µm]	*p* value
Mesio-distal	-6 ± 4	0.007*
Bucco-oral	-15 ± 7	< 0.001*
Apico-occlusal	-14 ± 5	< 0.001*
Total	-24 ± 7	< 0.001*
Rotational deviations (*n* = 822)	Difference between graduated and non-graduated dental staff value ± *CI* [°]	*p* value
Bucco-oral tipping	-0.11 ± 0.04	< 0.001*
Mesio-distal tipping	-0.03 ± 0.03	0.063
Mesio-distal rotation	-0.04 ± 0.05	0.069
Total	-0.13 ± 0.04	< 0.001*

^*^Significant difference (*p* < 0.05)

## Discussion

IO scanners generate dental-arch models by combining 3D images of small dental-arch segments to create a full-arch image. This requires the superimposition of overlapping segmental surfaces, which can lead to inaccuracies in the positions of small segments and individual teeth. Several previous studies investigated the precision or accuracy of IO scans by superimposing them onto reference desktop scans and topographically evaluating the deviations based on color-coding [3–7, 9, 13, 18]. Other studies evaluated the trueness of IO scans by measuring metric distances between corresponding model surface points [19–25]. The approach developed for the present study can be regarded as an enhancement of this latter technique, because the deviations of individual teeth are considered in all six degrees of freedom, i.e., 3D translational and rotational components.

In this study, desktop scans of resin models were used for reference. We investigated the median differences of single teeth in repeated desktop scans of one of the models (B) which revealed values of 6 µm (mesio-distal), -12.8 µm (bucco-oral), -9 µm (vertical), and 18 µm (total translation). These values can be considered clinically irrelevant at least for orthodontic applications. Therefore, one single desktop scan per model was used for reference. We evaluated the reliability of the whole superimposition procedure including the placement of the FA points in a pilot study. Here, differences between individual tooth positions determined by two different raters of 0.0021 µm (total translation) and 0.025° (total rotation) respectively, which has been considered negligible, as well. Therefore, data recorded by one single investigator (FK) were used for further processing.

**Fig. 7:**
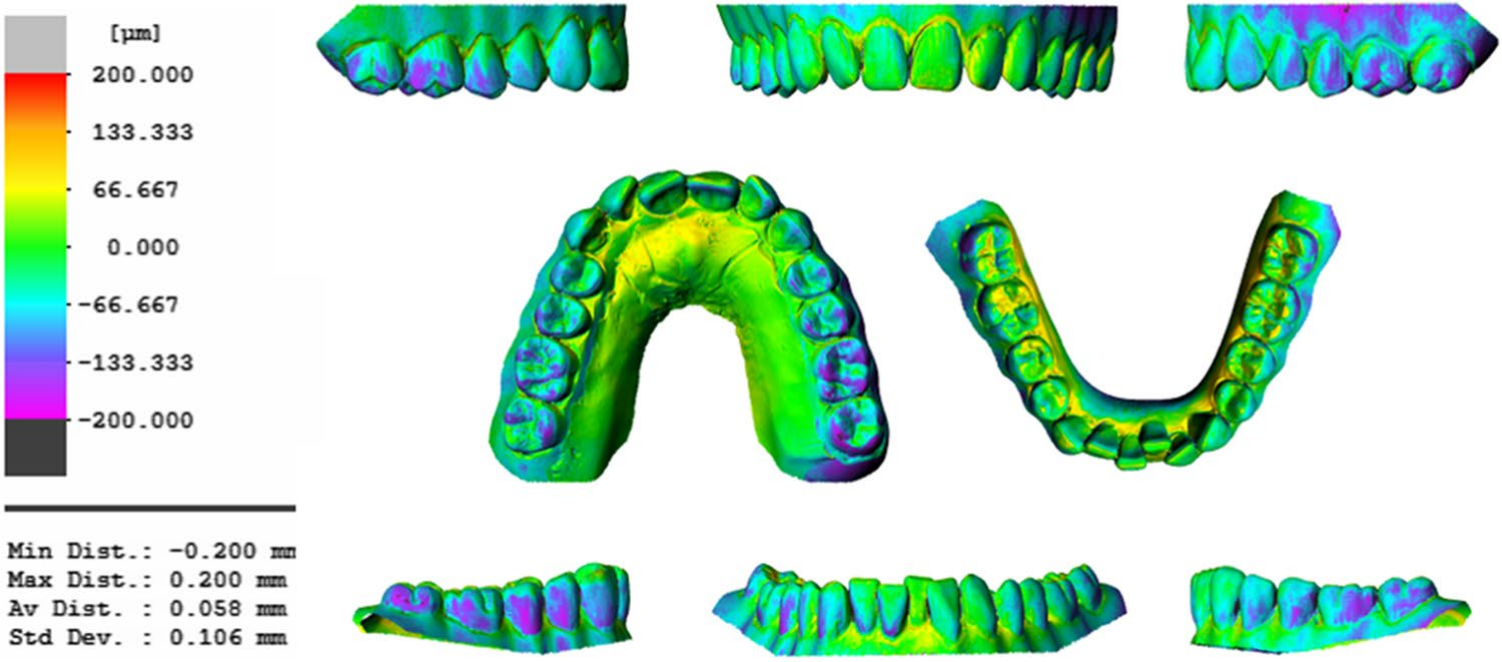
Typical example of a mesh comparison of an intraoral scan with the reference desktop scan, showing good agreement on the occlusal surfaces and incisal edges and increasingly large discrepancies on the distal surfaces of the lateral teeth

We generally observed that the translational (i.e., metric) deviations between IO scans and reference scans were quite small: The pooled mixed-effects model for all (mal)positions, individual teeth, raters and repetitions estimated mesial, oral, and intrusive displacements of 32, 76, and 58 µm, respectively. This pattern of individual tooth deviations suggests that the shape of the intraorally scanned dental-arch model tends to be slightly smaller than that of the reference model in both the bucco-oral and mesio-distal direction. Superimposition of the scans showed that this effect is most noticeable on the second molar crowns, i.e., at the distal ends of the dental arches. More specifically, on the IO scans, these teeth showed deviations of 45–91 µm and -57 to -111 µm in the mesial and oral directions, respectively. In contrast to previous studies, our study also determined rotational deviations of individual teeth in relation to the center of the labial crown (FA points). To the best of our knowledge, rotational deviations at a single-tooth level have not been considered before. The most consistent finding in our data was a rotation around the x-axis signifying labial or buccal tipping of both the anterior and posterior teeth. An example of this tendency is depicted in Fig. 7, which shows a very good match between the occlusal surfaces and incisal edges of the IO and reference scans (indicated in green) and larger deviations in the cervical region of the crowns. This might be due to the focus of the scanning software placed on fitting occlusal and incisal surfaces during the scanning process, which requires compensation of the smaller mesio-distal and transversal arch dimensions by means of buccal and labial tipping of the crowns. In addition, the buccal and labial tipping is consistent with the apical displacement of the FA points on buccal tooth surfaces.

The trueness of IO scans has already been evaluated in several previous in vitro and in vivo studies. For in vitro evaluations, scanning of master models either with desktop or industrial scanners and linear measurements between specific reference points or surfaces [20, 23, 26–29], determination of the deviations between nearest points of surface meshes after best-fit superimposition or visual inspection of color-coded images [9, 30] were common approaches. Tomita et al. [23] reported reduced intermolar widths of -50.9 µm (maxilla) and -113.6 µm (mandible) when comparing Trios IO scans with corresponding reference values. Their sagittal deviations indicated a shortening of the dental arches up to − 45.8 µm in three of the four quadrants. Other studies evaluated absolute deviations of the inter-molar distance to describe the transversal discrepancies in the posterior part of the virtual dental arch models. Three of these studies also using the Trios scanner system reported deviations in the range of 69 µm [30], 103 µm [27], or 165 µm [29] which is comparable to the decrease of the transversal second molar distance of -168 µm and -213 µm observed in the present study for the upper and lower arch, respectively. Mueller et al. [12] compared the surface meshes of superimposed IO scans and virtual reference models and found mean absolute deviations between both surface meshes of 17.1 and 26.8 µm depending on the scanning strategy applied. Their color-coded images also revealed the largest deviations in the horizontal dimension of the posterior dental arch with a constriction of the IO scans of ca. 100 µm.

Three studies evaluated trueness of IO scans at the level of single teeth [14, 31, 32]. It has to be noted, however, that only translational deviations were determined. Grünheid et al. [14] investigated extraoral scans of plaster casts using a Lava COS IO scanner and a desktop scanner and evaluated their scans similar to our approach. Reported single-tooth displacements in Lava COS scans of the upper arch indicated increasing buccal tooth displacements in posterior direction leading to an increase of the intermolar width of 170 µm. In the lower arch, however, a lingual molar displacement was observed resulting in a reduced intermolar width (-100 µm). Moreover, they reported a shortening of both the upper (-250 µm) and lower arches (-70 µm).

A more recent in vitro study by Vàg et al. [31] investigated single-tooth deviations on scans of PMMA master models using a PlanScan IO scanner and applying different scanning paths. They observed relatively large molar displacements in buccal direction leading to a transversal posterior widening of the upper (0.25–0.75 mm) and lower dental arches (0.5–1.1 mm), respectively. Single-tooth displacements were specified as absolute mean translational deviations determined at specific reference points. These deviations were 300 ± 9 µm which is clearly larger than the total translations observed in the present study for Trios scans.

The in vitro study by Nagy et al. [32] applied different IO scanning systems to determine the trueness of IO scans from a dissected maxilla of a human cadaver. IO scans and reference models were superimposed at the tooth which was scanned first which is principally different from the best-fit model superimposition applied in most studies (and in the present study, as well). This difference may explain the higher mean total translational deviations of individual teeth of 156 ± 8 µm observed by Nagy et al. [32] when compared to the value of 114 µm determined in the present study.

As mentioned, rotational deviations of single teeth in IO scans have not yet been quantitatively analyzed in vitro. Some previous studies, however, already investigated angular distortions of full-arch IO scans. For instance, Güth et al. and Keul and Güth [20, 26] analyzed the divergence of the lateral surfaces of a reference structure positioned on second molars. Corresponding angular deviations ranged between 0.003° and 0.73°. The magnitude of these values is roughly comparable with our pooled values of 0.58° for buccal tipping, 0.04° for mesial tipping, and 0.14° for distorotation.

In the context of in vivo studies, Grünheid et al. [14] reported single-tooth displacements in Lava COS IO scans compared to impression reference scans. In contrast to the findings of our in vitro study, they observed buccal displacements of posterior teeth. Corresponding values for the maxillary and mandibular second molars were 210 µm and 110 µm, respectively. This exemplifies that results of in vitro and in vivo studies of IO scans may be contrasting. In another in vivo study, Kuhr et al. [22] precisely positioned four metal spheres on the lower dental arch of 50 subjects using a transfer aid. The deviations of inter-sphere distances on Cara Trios IO scans were the largest in the transversal dimension of the posterior arch (97 ± 77 µm, absolute value). These findings were recently confirmed by Kwon et al. [33] using an industrial scanner for directly generating a highly accurate virtual reference model of the patients’ dental arches. Again, the largest deviations were observed in the transversal dimension of the posterior lower arch with a mean absolute deviation of 96.8 ± 43.4 µm for the Trios 3 IO scanner. Reported values for overall trueness were 51.0 ± 37.8 µm. A similar approach for determination of reference models was applied by Nedelcu et al. [34] who found an expansion of ca. 25 µm per side in the premolar region of Trios full-arch IO scans which is contradictory to the posterior arch constriction determined in in vitro investigations such as the present study.

With regard to the trueness of IO scans of different models of tooth (mal)positions, it has to be noted that the variety of tooth irregularities and malocclusions in real patients is much wider than those reflected by the three model pairs included. The selected tooth malposition types, however, reflect morphological features that actually present typical difficulties for an IO scanner, such as overlapping tooth crown surfaces with narrow contact regions and inclined crown surfaces both leading to various distances to the scanner head (model B), or interdental spaces leading to “holes” in the surfaces to be scanned (model C). Since IO scans from other malocclusion types are probably confronted with similar challenges, one might speculate that the trueness of corresponding IO scans may be reasonably comparable with those reported here. For the three investigated model pairs, trueness was the highest for the model with anterior crowding (model B), and was the poorest for the models with regular front teeth. This corresponds to the findings of Anh et al. [4], who investigated the effect of tooth irregularities on the precision of Trios scans and also observed the largest deviations among repeated scans of an ideal arch model. One possible explanation for the relatively poor performance when scanning aligned and regular teeth might be the lack of surface characteristics. Spaces and anterior crowding provide additional and more complex 3D geometries that can contribute to a more exact 3D stitching of the captured individual images during the scanning process. These geometries are either tooth edges between closely packed front teeth, or distinct mesial or distal surfaces in flared or spaced frontal or lateral teeth. These findings are in accordance with those of Rudolph et al. [35], who found that steep and parallel opposing tooth surfaces in incisor preparations are usually the most difficult areas to capture for most digitizing systems. Because the surfaces of prepared anterior teeth have a similar angle to that of unprepared incisors, it is likely that these results can be transferred to the regular and aligned dental arch.

Estimated *SD* for translational deviations of individual tooth positions among repeated scans within raters and among single scans of different raters are ≤ 53 µm. It might be speculated that that the smaller *SD* values for intraobserver variability are related to the influence of the individual handling of the scanner during the scanning procedure leading to smaller deviations among repeated scans within one person. Generally, *SD* for intra- and interobserver variability are similar to the precision reported by most in vitro studies using Trios scanners [4, 23, 28, 29]. Slightly smaller precision values were reported by Müller et al. [12], with mean superimposition differences in the range of 7.9 ± 5.6 µm and 35.0 ± 51.1 µm among repeated scans performed by one investigator. Their lower values might be related to the exclusion of the outliers [36]. In contrast, Renne et al. [30] found considerably higher mean *SD* of 105.6 µm among scans of different investigators using the Trios 3 scanner. in vivo studies using Trios scanners reported precision values between 43 [18] and 52 µm [7] which are also in the range of those observed in the present study. Estimated total rotational deviations of individual tooth positions among repeated scans within and between raters are ≤ 0.25° which may also be regarded as negligible in the context of orthodontic applications. Also Güth et al. [20] and Keul and Güth [26] reported in vitro and in vivo precision via the *SD* of angular measurements ranging between 0.04° and 0.15°. It has to be noted, however, that their values represent angular deviations between lateral planes of a specific reference device positioned between second molars, which are not quite comparable to angular single-tooth deviations.

Comparison between the trueness of IO scans performed by dentists and non-graduate investigators with similar level of routine revealed very small differences which may be regarded as negligible in clinical respects. Based on this finding, one might speculate that the operator’s level of education is not an important issue in this context. It is important to note, however, that this should not be interpreted to mean that IO scanning can be completely delegated to non-graduate dental staff members. More concretely, it is the clinician to decide in view of the subsequent application of the digital model whether the quality of the IO scan comprising the dental arch and the adjacent gingival tissues is sufficient or not. Furthermore, only the clinician who performed the functional examination of the patient may judge whether the registration of the jaws actually reflects the patient’s habitual lower jaw position in a correct manner. This means that the final responsibility for the IO scan of the dental arches and intermaxillary relation lies with the dentist who has to guarantee the sufficient quality, correct documentation and the fulfilment of statutory requirements.

Study models are most suitable to evaluate the trueness and precision of IO scans, because the most accurate reference models are those obtained from desktop or micro-CT scans, which cannot be performed of the dental arches in situ. We used resin casts made from denture acrylic with denture teeth to simulate natural color and reflection properties. The disadvantage of the resin model was the need to use powder for the reference scans taken with the desktop scanner. Pilot studies measuring the thickness of powder after repeated applications showed a layer of 15.5 µm (± 2.6), which is below the precision of the desktop scanner guaranteed by the manufacturer [16]. A clear advantage of our method is that it does not require the attachment of any additional objects to the dental arch or reference model as described in other studies [22, 28, 29]. It has to be noted, however, that the “IO scans” evaluated in this in vitro study were generated in a simulated intraoral environment. Although the setting during the scanning procedure—in particular, this means the geometric interrelations between the scanning device and the object—might have been quite realistic, we assume that the results cannot be directly transferred to the real clinical situation. This is due to additional influencing factors present in in vivo (such as the tongue and saliva and/or small deformations of the mandible during jaw opening) that might compromise the scanning accuracy in patients.

## Clinical relevance

The results of this study suggest that the trueness of 3D digital models obtained from Trios 3 IO scans is sufficiently high and intra- and inter-examiner reproducibility are acceptable for orthodontic diagnostic purposes. Objective grading systems such as the American Board of Orthodontics Grading system [37] and Peer Assessment Rating index [38] define misalignments as deviations of more than 0.5 mm and 1 mm, respectively. This means that IO scans are sufficiently accurate to detect such misalignments.

Regarding the use of digital dental-arch models to manufacture orthodontic appliances as an extension to a complete computer-aided-design and computer-aided-manufacturing (CAD/CAM) process, other factors must be considered, e.g., errors during the model printing process. Furthermore, the use of digital arch models might differ between elastic and rigid appliances. Full-arch IO scans could also be applied in aligner therapy. Depending on the aligner system, correction steps of 0.2 mm or more are used, which is larger than the highest median inaccuracy found in our study. Problems with aligner fit are more likely to occur when data is used to treat aligner patients with anterior flaring or for the fabrication of cast appliances, and a defined direction of insertion is essential.

## Conclusions

- In the superimposition and measurement method described, trueness, as well as intra- and inter-examiner reproducibility of virtual models obtained by means of full-arch IO scans, can be quantified in terms of the deviation of each tooth in all three planes of space, i.e., translation along and rotation around three spatial axes.
- In the virtual models derived from IO scans, teeth are only slightly displaced to the oral side and slightly tipped buccally, resulting in very good agreement between the occlusal and incisal surfaces of IO and reference scans.
- In daily clinical routine, IO scanning of dental arches can be integrated into the workflow of non-graduate dental staff. By controlling the level of routine, the level of education does not seem to affect the trueness of the digital model derived.
- Deviations between IO and reference scans are small, and trueness, as well as reproducibility within and between examiners, can be regarded as clinically acceptable for diagnostic purposes and computer-aided manufacturing of removable orthodontic appliances such as aligners.

## References

[1] Allen KL, Schenkel AB, Estafan D (2004). An overview of the CEREC 3D CAD/CAM system. Gen Dent.

[2] International Organization for Standardization (2012) Accuracy (trueness and precision) of measurement methods and results - Part 1: General principles and definitions. 03.120.30(ISO 5725–1:1994). https://www.iso.org/standard/11833.html. Accessed 30 May 2018

[3] Flügge TV, Schlager S, Nelson K (2013). Precision of intraoral digital dental impressions with iTero and extraoral digitization with the iTero and a model scanner. Am J Orthod Dentofacial Orthop.

[4] Anh J-W, Park J-M, Chun Y-S (2016). A comparison of the precision of three-dimensional images acquired by 2 digital intraoral scanners: effects of tooth irregularity and scanning direction. Korean J Orthod.

[5] Gan N, Xiong Y, Jiao T (2016). Accuracy of intraoral digital impressions for whole upper jaws, including full dentitions and palatal soft tissues. PLoS ONE.

[6] Kamimura E, Tanaka S, Takaba M (2017). In vivo evaluation of inter-operator reproducibility of digital dental and conventional impression techniques. PLoS ONE.

[7] Lim J-H, Park J-M, Kim M (2017). Comparison of digital intraoral scanner reproducibility and image trueness considering repetitive experience. J Prosthet Dent.

[8] Patzelt SBM, Bishti S, Stampf S (2014). Accuracy of computer-aided design/computer-aided manufacturing-generated dental casts based on intraoral scanner data. J Am Dent Assoc.

[9] Patzelt SBM, Emmanouilidi A, Stampf S (2014). Accuracy of full-arch scans using intraoral scanners. Clin Oral Investig.

[10] Zimmermann M, Koller C, Rumetsch M (2017). Präzision von Guided-Scanning-Verfahren bei digitalen Gesamtkieferabformungen in vivo (Precision of guided scanning procedures for full-arch digital impressions in vivo). J Orofac Orthop.

[11] Kim J, Park J-M, Kim M (2016). Comparison of experience curves between two 3-dimensional intraoral scanners. J Prosthet Dent.

[12] Müller P, Ender A, Joda T (2016). Impact of digital intraoral scan strategies on the impression accuracy using the TRIOS Pod scanner. Quintessence Int.

[13] Zimmermann M, Koller C, Rumetsch M (2017). Precision of guided scanning procedures for full-arch digital impressions in vivo (Prazision von Guided-Scanning-Verfahren bei digitalen Gesamtkieferabformungen in vivo). J Orofac Orthop.

[14] Grünheid T, McCarthy SD, Larson BE (2014). Clinical use of a direct chairside oral scanner: an assessment of accuracy, time, and patient acceptance. Am J Orthod Dentofacial Orthop.

[15] Vogel AB, Kilic F, Schmidt F (2015). Optical 3D scans for orthodontic diagnostics performed on full-arch impressions. Completeness of surface structure representation. J Orofac Orthop.

[16] Vogel AB, Kilic F, Schmidt F (2015). Dimensional accuracy of jaw scans performed on alginate impressions or stone models: A practice-oriented study. J Orofac Orthop.

[17] Schmidt F, Kilic F, Piro NE (2018). Novel method for superposing 3D digital models for monitoring orthodontic tooth movement. Ann Biomed Eng.

[18] Ender A, Attin T, Mehl A (2016). In vivo precision of conventional and digital methods of obtaining complete-arch dental impressions. J Prosthet Dent.

[19] Ender A, Mehl A (2015). In-vitro evaluation of the accuracy of conventional and digital methods of obtaining full-arch dental impressions. Quintessence Int.

[20] Güth J-F, Edelhoff D, Schweiger J (2016). A new method for the evaluation of the accuracy of full-arch digital impressions in vitro. Clin Oral Investig.

[21] Kim J-H, Kim K-B, Kim W-C (2014). Accuracy and precision of polyurethane dental arch models fabricated using a three-dimensional subtractive rapid prototyping method with an intraoral scanning technique. Korean J Orthod.

[22] Kuhr F, Schmidt A, Rehmann P (2016). A new method for assessing the accuracy of full arch impressions in patients. J Dent.

[23] Tomita Y, Uechi J, Konno M (2018). Accuracy of digital models generated by conventional impression/plaster-model methods and intraoral scanning. Dent Mater J.

[24] Wiranto MG, Engelbrecht WP, Tutein Nolthenius HE (2013). Validity, reliability, and reproducibility of linear measurements on digital models obtained from intraoral and cone-beam computed tomography scans of alginate impressions. Am J Orthod Dentofacial Orthop.

[25] Ender A, Zimmermann M, Mehl A (2019). Accuracy of complete- and partial-arch impressions of actual intraoral scanning systems in vitro. Int J Comput Dent.

[26] Keul C, Güth J-F (2020). Accuracy of full-arch digital impressions: an in vitro and in vivo comparison. Clin Oral Investig.

[27] Celeghin G, Franceschetti G, Mobilio N et al (2021) Complete-arch accuracy of four intraoral scanners: an in vitro study. Healthcare (Basel) 9. 10.3390/healthcare9030246 10.3390/healthcare9030246 PMC8002151 33804310

[28] Muallah J, Wesemann C, Nowak R (2017). Accuracy of full-arch scans using intraoral and extraoral scanners: an in vitro study using a new method of evaluation. Int J Comput Dent.

[29] Wesemann C, Muallah J, Mah J (2017). Accuracy and efficiency of full-arch digitalization and 3D printing: a comparison between desktop model scanners, an intraoral scanner, a CBCT model scan, and stereolithographic 3D printing. Quintessence Int.

[30] Renne W, Ludlow M, Fryml J (2017). Evaluation of the accuracy of 7 digital scanners: an in vitro analysis based on 3-dimensional comparisons. J Prosthet Dent.

[31] Vág J, Nagy Z, Simon B (2019). A novel method for complex three-dimensional evaluation of intraoral scanner accuracy. Int J Comput Dent.

[32] Nagy Z, Simon B, Mennito A (2020). Comparing the trueness of seven intraoral scanners and a physical impression on dentate human maxilla by a novel method. BMC Oral Health.

[33] Kwon M, Cho Y, Kim D-W (2021). Full-arch accuracy of five intraoral scanners: in vivo analysis of trueness and precision. Korean J Orthod.

[34] Nedelcu R, Olsson P, Nyström I (2018). Accuracy and precision of 3 intraoral scanners and accuracy of conventional impressions: a novel in vivo analysis method. J Dent.

[35] Rudolph H, Salmen H, Moldan M (2016). Accuracy of intraoral and extraoral digital data acquisition for dental restorations. J Appl Oral Sci.

[36] Ender A, Mehl A (2013). Accuracy of complete-arch dental impressions: a new method of measuring trueness and precision. J Prosthet Dent.

[37] The American Board of Orthodontics (2012) Grading System for Dental Casts and Panoramic Radiographs. https://www.americanboardortho.com/media/1191/grading-system-casts-radiographs.pdf. Accessed 28 Feb 2018 10.1016/s0889-5406(98)70179-9 9810056

[38] Richmond S, Shaw WC, Roberts CT (1992). The PAR Index (Peer Assessment Rating): Methods to determine outcome of orthodontic treatment in terms of improvement and standards. Eur J Orthod.

